# Understanding Adults’ Spatial Cognitive Processes: A Time-Embedded N-Grams Model with Machine Learning

**DOI:** 10.3390/jintelligence14060105

**Published:** 2026-06-10

**Authors:** Qiwei He, Yiming Chen, Elizabeth L. Tighe, Gal Kaldes

**Affiliations:** 1 Department of Psychology and Data Science and Analytics Program, Georgetown University, Washington, DC 20007, USA; 2 Department of Educational Psychology, University of Minnesota, Twin Cities, MN 55455, USA; chen9462@umn.edu; 3 Department of Psychology, Georgia State University, Atlanta, GA 30303, USA; etighe@gsu.edu; 4 Adult Literacy Research Center, Department of Learning Sciences, Georgia State University, Atlanta, GA 30303, USA; gkaldes1@gsu.edu

**Keywords:** spatial cognitive processes, time-embedded *n*-grams, machine learning, numeracy, educational assessment, PIAAC

## Abstract

Technological advances have transformed the landscape of educational assessments, particularly in how we collect and analyze data on individuals’ cognitive processes and interactions with assessment items. The rich data recorded in log files during human–machine interactions are often referred to as process data. This study uses sequential process data from the 2012 Program for the International Assessment of Adult Competencies (PIAAC), focusing specifically on how respondents navigate an interactive numeracy item (“Map”) that involves spatial cognition. The objectives of this study are trifold: (1) to explore factors that contribute to success or failure on a spatial numeracy item, (2) to identify spatial cognitive process features across high and low numeracy performance levels, and (3) to introduce a novel time-embedded *n*-grams model to incorporate elapsed time with sequential actions in process data analysis. Using a sample of 596 U.S. adult respondents, we employed the time-embedded *n*-grams model and two machine learning methods, random forest and XGBoost, to predict respondents’ numeracy performance and to identify robust classifiers. Results indicate that time-related features and understanding directions in the spatial dimensions are the most predictive of adults’ numeracy skills. The findings highlight the potential of process data to support analyses of latent numeracy skills and cognitive processes in educational contexts.

## 1. Introduction

Spatial cognition plays a vital role in everyday functioning and serves as a foundational skill for success in scientific, technological, and problem-solving domains (Z. C. K. Hawes et al., 2022; Klencklen et al., 2012; Montello & Raubal, 2013). It involves reasoning about spatial relationships, such as distance, angles, and directions, and supports interpretation of symbolic representations, including maps, diagrams, and models (Vasilyeva & Lourenco, 2012). These abilities enable individuals to navigate and make sense of complex environments.

To better understand how these skills support real-world navigation and problem-solving, spatial cognition can be conceptualized as comprising several interrelated processes, including visuospatial perception, mental imagery, spatial memory, and navigation (Burgess, 2008; Klencklen et al., 2012). Visuospatial perception supports the interpretation of spatial relationships, including categorical (abstract) and coordinate (metric) representations (Kolb & Whishaw, 2009; Reese & Stiles, 2005; van der Ham & Borst, 2011), while mental imagery involves operations such as mental rotation and scanning that contribute to the construction of cognitive maps. Spatial memory underpins these processes by supporting the retention of object locations and spatial contexts (Ruggiero et al., 2008), and navigation integrates them to enable wayfinding and route planning, often coordinating between either egocentric (self-centered) or allocentric (environment-centered) frames of reference (Moffat, 2009).

Despite its importance, measuring spatial cognitive skills presents significant methodological challenges. Traditional psychometric instruments, such as the Mental Rotation Test (MRT; Hegarty, 2018; Maeda & Yoon, 2013), mirror image recognition (Keromnes et al., 2019), and embedded figure tasks (Glicksohn & Kinberg, 2009), offer standardized assessments of spatial visualization, but often capture only isolated components of spatial ability and lack ecological validity. In contrast, real-world and contextual tasks, including maze navigation, route planning, landmark recall, and block construction, provide more ecologically valid insights into how spatial cognition operates in applied settings. Recent innovations, such as virtual reality simulations, eye-tracking, and motion capture, further expand the possibilities for immersive and dynamic measurement (Kiefer et al., 2017; S. Wang et al., 2024). However, these approaches often suffer from limited psychometric validation, small sample sizes, and context sensitivity, which constrain their generalizability and scalability (Man, 2024).

**Figure 1:**
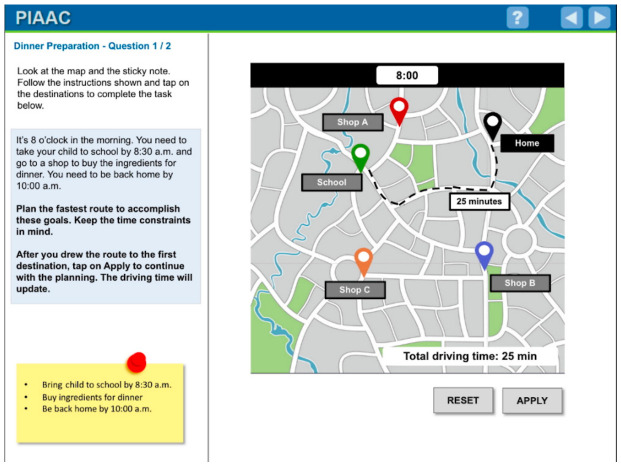
An example item “Dinner Preparation” with map content in PIAAC 2022 (OECD, 2021, p. 163). *Note.* At the upper-right corner, the question-mark icon indicates the Help function. The left and right arrow icons indicate the Previous Item and Next Item navigation buttons.

One way to address these limitations is through large-scale assessments, where spatial cognition can be examined at scale. In these settings, spatial cognition is often operationalized within numeracy tasks that require integration of spatial and quantitative information (OECD, 2012). International large-scale educational assessments have increasingly incorporated spatial navigation tasks to measure abilities in dimension and shape. For example, route planning in the Programme for International Student Assessment (PISA) 2012, map recognition in the Programme for the International Assessment of Adult Competencies (PIAAC) 2012, and adaptive problem-solving (APS) in PIAAC 2022 require geographic reasoning and strategic coordination to determine optimal navigation paths. Figure 1 shows an example APS task in PIAAC 2022 involving map navigation across multiple locations. These assessments provide large-scale data to examine spatial cognition in real-world task contexts, supported by nationally representative samples and rich background data.

Beyond their scale, digital assessment platforms enable the collection of process data, which are detailed logs of how individuals interact with tasks in real time. These data provide granular insights into the cognitive strategies individuals employ and allow researchers to trace patterns of success and failure at the individual level (Goldhammer et al., 2017; Q. He et al., 2021; S. He & Cui, 2025).

Building on this approach, the present study examines adults’ navigation patterns on a PIAAC interactive numeracy item referred to as “Map”. This task requires respondents to mentally simulate spatial orientation to generate a series of directional paths. Using process data and machine learning techniques to model sequential task interactions, we aim to identify key factors that contribute to success or failure on the Map task. In doing so, this study addresses a key gap in large-scale investigations of adult spatial cognition and contributes to the development of more valid, scalable, and context-sensitive assessment frameworks.

### 1.1. Embodied Cognition Theories

The Map task requires respondents to mentally simulate orientation and movement through space, making it a useful context for examining how spatial reasoning is grounded in bodily experiences. This perspective aligns with embodied cognition theories, which propose that abstract concepts, including spatial and numeric relationships, are shaped by sensorimotor processes (Barsalou, 1999; Wilson, 2002). Rather than treating spatial cognition as purely abstract, this framework emphasizes the role of perception and action in structuring spatial understanding. Evidence from this perspective includes the “mental number line”, in which numbers are spatially represented from a left-to-right continuum (Dehaene et al., 1993). Similarly, research has shown that gesturing (hand movements) improves performance on spatially demanding numerical tasks by supporting mental simulation and visuospatial working memory processes (Aldugom et al., 2020; Brooks et al., 2018).

A concrete example is provided by C. Zhu et al. (2023), who used data-driven thematic analysis to identify themes relevant to spatial thinking based on multimodal evidence, including physical artifacts, classroom interactions, and recorded problem-solving processes. Their findings showed that participants generated diverse strategies for constructing visual-spatial representations, such as mapping quantities onto tangible objects, arranging materials in spatial order, and unitizing elements to establish visual parameters. Participants also iteratively refined their understanding as they moved between abstract numerical values and tangible materials during the embodied making process. These findings illustrate that spatial reasoning is not only shaped by embodied processes but can also be observed through individuals’ interactions with tasks. This perspective aligns with process data approaches, which capture how such cognitive processes unfold over time. Large-scale assessments that record both performance and process data therefore provide a unique opportunity to examine these mechanisms in real-world contexts.

### 1.2. Assessing Numeracy Skills in PIAAC

PIAAC is a large-scale international assessment designed to evaluate the literacy, numeracy, and problem-solving skills of the working-age population (ages 16 to 65). Administered in technology-rich environments, PIAAC tasks often require individuals to engage with complex, real-world scenarios, providing a relevant context for examining how cognitive skills are applied in practice. Countries use PIAAC data to evaluate how well adults are prepared to participate in civic, cultural, and economic life. Beyond core cognitive domains, PIAAC collects detailed background and interaction data, enabling researchers to examine both the distribution of adult skills and the processes underlying task performance. As one of the most comprehensive assessments of adult competencies, PIAAC offers a scalable and policy-relevant platform for investigating how individuals engage with cognitively demanding tasks (Curry, 2019).

Within PIAAC, numeracy is defined as “the ability to access, use, interpret, and communicate mathematical information and ideas in order to engage with and manage the mathematical demands of everyday adult life” (OECD, 2012, p. 36). The numeracy framework conceptualizes numeracy through four interrelated facets: (1) contexts, (2) mathematical content, (3) cognitive strategies, and (4) representations of information. Together, these facets shape how individuals engage with assessment tasks. In the Map task analyzed in this study, these facets are reflected in distinct but overlapping ways. The task is situated in an applied context that requires respondents to navigate a real-world scenario, while the mathematical content is primarily drawn from the dimension and shape domain, involving spatial reasoning, orientation, and the interpretation of two-dimensional representations. Respondents must also employ cognitive strategies, including identifying relevant information and evaluating possible routes. Information is presented through visual and spatial representations within a digital interface, requiring individuals to coordinate perceptual and spatial reasoning processes. Together, these facets provide a framework for understanding how spatial cognition and navigation behavior are expressed within the process data.

### 1.3. Gaps in Adults’ Digital Numeracy Skills

Despite the comprehensive framework used to assess numeracy in PIAAC, substantial gaps remain in adults’ digital numeracy skills. PIAAC defines numeracy across six proficiency levels (Below Level 1-Level 5), with meeting proficiency defined as Level 3^1^. However, many U.S. adults fall below proficiency thresholds (57% at or Below Level 2 in 2023 Cycle 2; 52% at or Below Level 2 in 2012–2017 Cycle 1; National Center for Education Statistics, 2019, 2024), suggesting that a large proportion of adults are still developing skills related to “accessing, using, and reasoning critically with mathematical content” (OECD, 2021, p. 19) in daily life. These challenges are further compounded by the increasing reliance on digital assessment environments, which demand not only literacy and numeracy proficiency but also the ability to navigate interactive interfaces, including selecting, interpreting, and acting on information within dynamic tasks (Brinkley-Etzkorn & Ishitani, 2016; Graesser et al., 2019). As emphasized in the PIAAC framework, numeracy extends beyond procedural skills to include reasoning in real-world contexts. This suggests a more use-oriented, applied approach as opposed to decontextualized math skills and procedural (step-like) math (Curry, 2019).

Despite the high stakes associated with digital assessment outcomes, limited attention has been paid to how adults, particularly those with lower skills, engage with these platforms. As computer-based assessments increasingly capture granular process data (e.g., time-stamped action sequences, navigation paths), researchers are now positioned to examine not just whether an answer is correct, but *how* and *when* it was attempted or solved (Goldhammer et al., 2013; Q. He et al., 2023a). These data make it possible to analyze behaviors such as item skipping, time allocation, and response efficiency, which are critical for interpreting performance and identifying barriers to success. Recent studies have shown that such behavioral indicators are particularly well-suited for capturing individuals’ problem-solving strategies and patterns of engagement (e.g., Q. He et al., 2021; Zhang et al., 2024), offering a more nuanced view of how respondents interact with tasks beyond what response accuracy alone can convey.

Within this context, the present study focuses on spatial cognition, which is an often-overlooked component of digital numeracy. Spatial cognition supports tasks such as interpreting graphs, navigating interfaces, and mentally manipulating quantities. In digital assessment environments, these processes are reflected in observable behaviors, including how adults allocate time, sequence actions, and visually navigate numeracy items. By examining these behavioral patterns, we aim to uncover the cognitive strategies that underlie success or failure among adults with different numeracy skill levels (higher/lower). These insights will inform targeted instructional approaches to strengthen spatial navigation and digital task fluency among underperforming groups. Understanding these patterns requires data that capture how actions unfold and how responses are paced during task completion.

### 1.4. Sequential Process Data Analysis

Computer-based assessment platforms capture detailed log files that allow researchers to examine how individuals interact with tasks in real time. These granular records, commonly referred to as *process data*, document every action a respondent takes on screen, including mouse movements, time spent on tasks, navigation paths, and response revisions. Typically stored as ordered sequences of multi-type, time-stamped events, process data provides insights into when and how individuals engage with digital tasks (Goldhammer et al., 2014; Guo et al., 2016; Kaldes et al., 2024; M. Zhu & Feng, 2015). These records reflect both the sequence of actions and the timing of those actions, making it possible to examine how behavior unfolds during task engagement. The relationship between action and timing is critical, as delays, pauses, and transitions can reflect underlying cognitive processes such as planning, uncertainty, or strategy shift.

A central challenge in process data analysis is how to represent both the sequence of actions and the timing of those actions in a way that preserves behavioral structure while remaining suitable for statistical and machine learning models. Existing approaches address this challenge in different ways but mainly capture only part of the underlying structure of sequential task behavior because they treat action sequences and timing information independently (Q. He et al., 2023a). It is an issue evident in feature-based methods such as n-gram sequence analysis, which extract localized behavioral patterns from contiguous subsequences for predictive modeling while still separating actions from their temporal dynamics (Y. Chen et al., 2019; Han et al., 2019; Q. He & von Davier, 2016; Liao et al., 2019; Salles et al., 2020; Stadler et al., 2019).

In contrast, sequence distance methods quantify similarity across entire sequences (Dong & Pei, 2007), for example, using the Longest Common Subsequence (Q. He et al., 2021; Ulitzsch et al., 2021) or Dynamic Time Warping (Q. He et al., 2023a), capturing global structure but lacking compatibility with feature-based modeling frameworks. Model-based approaches, such as Hidden Markov Models (HMMs), use latent sequence modeling to infer underlying cognitive states from observed action patterns, providing insight into latent structure but relying on strong assumptions and not directly preserving observable temporal dynamics (Tang, 2024; Tenison & Arslan, 2020; Xiao et al., 2021). More recently, artificial intelligence-supported approaches have been explored (Bulut et al., 2025), but process data differ substantially from the single-dimensional inputs used to train such models, and their relatively limited scale constrains their applicability in assessment contexts.

Among these approaches, gram-based methods provide a particularly strong foundation for modeling process data. Unlike sequence distance methods and model-based approaches such as HMMs (X. Liu et al., 2025; Tenison & Arslan, 2020; Xiao et al., 2021), n-gram representations offer a flexible, feature-based framework that can be directly integrated into statistical and machine learning analyses. In addition, n-grams capture localized patterns of behavior that are interpretable in terms of task actions, making them appropriate for identifying meaningful behavioral markers. Critically, their modular structure allows for the incorporation of additional information, such as temporal features, without fundamentally altering the representation. This flexibility makes n-grams particularly well-suited for extending sequence analysis to jointly model action sequences and timing information within a unified framework.

The present study builds on this foundation by introducing a novel time-embedded n-grams approach that addresses a key limitation of traditional gram-based methods, namely, the separation of action and timing into distinct dimensions. The time-embedded n-grams method incorporates elapsed time directly into the n-gram structure, reducing multidimensional sequences into a matrix-based representation that captures both navigation paths and pause durations between routing points. This integrated representation provides a more comprehensive view of how individuals engage with spatially demanding tasks in real time.

## 2. Aims and Research Questions

The present study examines how sequence-based behavioral patterns unveil adults’ spatial cognition process on the interactive numeracy task “Map” in PIAAC 2012. The granular process data allows us to investigate how action- and time-based patterns reflect underlying cognitive strategies and distinguish successful from unsuccessful performance.

This study focuses on three primary objectives: (1) to identify behavioral patterns associated with success and failure on a spatial numeracy item, (2) to compare process features across adults with different levels of numeracy proficiency, and (3) to introduce a time-embedded n-grams approach for modeling sequential process data that integrates action and timing information. To achieve these goals, we apply machine learning algorithms (Random Forest [RF] and Extreme Gradient Boosting [XGBoost]) to time-embedded n-gram representations to identify the most robust predictors of problem-solving success and numeracy skill level.

Specifically, this study addresses the following research questions:

RQ1. What behavioral patterns and strategies differentiate successful and unsuccessful attempts to solve a spatial numeracy task?

RQ2. Which process features most robustly predict adults’ numeracy proficiency?

## 3. Methods

### 3.1. Sample

The sample included 696 adult respondents from the U.S. PIAAC 2012 dataset who completed the numeracy Map item. Due to discrepancies between the process and response data files, 100 cases could not be matched and were excluded from subsequent analyses, resulting in a final sample size of *N* = 596. Among these participants, 54.9% self-identified as female. Based on the PIAAC numeracy proficiency scale, 296 individuals were categorized as having lower numeracy skills (Level 2 and below), while 300 were classified as having higher numeracy skills (Levels 3–5). Overall item performance indicated a correct response rate of 56.2% (335/596), with 43.8% (261/596) responding incorrectly.

Self-reported race/ethnicity distributions were: 72.2% White, 10.3% Black, 8.1% Hispanic, and 9.4% identifying as other, with two cases missing. The sample mean age was 39.02 years (*SD* = 13.96). Familiarity with information and communication technology (ICT) was assessed separately for home and workplace contexts. At home, the mean ICT was 2.26 (*SD* = 0.90; 46 missing), while at work it was 2.20 (*SD* = 1.09; 214 missing). Stratified by numeracy level, individuals in the higher numeracy group reported greater ICT familiarity (home: 2.47 vs. 2.04; work: 2.34 vs. 1.98). Detailed subgroup analyses, including item performance by numeracy classification, are presented in Table 1.

### 3.2. Instrument

**Figure 2:**
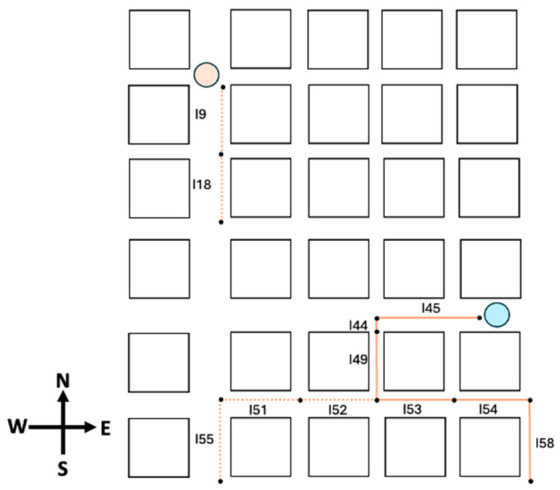
A demo of the Map item that mimics the symbolic structure of the original PIAAC task. The sequence of solid lines indicates the correct path; the sequence of dashed lines indicates the commonly incorrect paths. The blue marker indicates the correct starting point; the orange marker indicates a distractor starting point.

The Map item illustrates the integration of spatial cognition and spatial navigation within a real-world context. It requires respondents to interpret spatial layouts and directional cues while reasoning about movement and transformation. Figure 2 presents a demonstration of the Map item, designed to mimic the symbolic structure of the original PIAAC task.

In this item, participants are instructed to follow directional commands (e.g., “turn left,” “turn right,” “go straight”) to identify corresponding locations on the map and determine the correct route to a designated destination. Each viable path segment is labeled with an “I” prefix (e.g., I45, I49) to denote its position within the navigation sequence. A single click on the route signifies the “selection” of a path section, while clicking on an already selected path indicates its “deselection”. All the interactive actions with time stamps were captured for each respondent during the problem-solving process in PIAAC.

In Figure 2, the blue marker indicates the starting point, while the orange marker serves as a distractor. The correct route is depicted with a solid line, beginning at I45 and proceeding through I44 (optional), I49, I53, I54, and I58. Common incorrect paths are shown with dashed lines. Notably, the sequence in which segments are selected does not influence scoring; only the final route configuration is evaluated. Thus, whether a respondent clicks on I45 or I58 first is immaterial to correctness, though such choices may reflect strategic differences across respondent groups.

Successful completion of the item hinges on the respondent’s ability to mentally simulate their orientation within the spatial environment, rather than relying solely on the static visual representation of the map. A particularly illustrative example occurs at the intersection of I49, I52, and I53, where the instruction “turn left” is provided. To interpret this correctly, respondents must mentally position themselves as walking eastward and recognize that the intended “left” turn, relative to their orientation, is visually represented on the right side of the map. This mirror effect introduces a cognitively demanding element, requiring dynamic spatial transformation and embodied perspective-taking.

### 3.3. Time-Embedded n-Grams

As previously noted, He and von Davier (2016) introduced the n-grams method to segment long sequences into manageable mini-sequences, thereby facilitating the representation of sequential settings. This approach supports gram-based feature extraction and has demonstrated efficiency in process data analysis (Liao et al., 2019; Stadler et al., 2019; Ulitzsch et al., 2022; Zhang et al., 2024). However, traditional n-gram methods primarily focus on the sequence of actions, often neglecting the temporal dimension, which is not easily incorporated (Q. He & von Davier, 2016).

To address this limitation, Tenison and Arslan (2020) and Arslan et al. (2023) introduced a “pause” behavior between consecutive actions, using a fixed time threshold to distinguish between short and long pauses. These thresholds are typically determined based on researchers’ experience and are often applied uniformly across all action pairs. This uniformity may not reflect actual behavioral patterns, as the perception of pause duration is inherently relative. For instance, a 10 s interval may be considered a long pausing time for actions that typically occur in rapid succession, but relatively short for action pairs that usually involve extended cognitive processing.

To better capture this temporal nuance, we propose an alternative time-embedded n-grams method that incorporates the pause duration between each pair of actions, based on their empirical distributions. Specifically, two steps are required.

Step 1: This step filters action pairs to retain only those that occur with sufficient reliability, defined as appearing at least five times. In this study, we initially extracted 531 unique bigrams. To ensure reliability, we excluded those with a total frequency of five or fewer occurrences, yielding a refined set of 105 unique bigrams. This filtering step reflects the assumption that when action pairs occur infrequently, the elapsed time between them may not be a reliable indicator of behavioral patterns. Consequently, we did not assign a pause action in such cases. As a result, only the 105 action pairs meeting the frequency threshold were associated with elapsed times that qualified for adding the “pause” action in between.

Step 2: This step defines the ordinal categories of pause duration for each action pair. To embed temporal information, we introduced a new “pause action” into the original sequences. For each bigram, we examined the distribution of elapsed time and categorized pauses as follows: durations below the 25th percentile were labeled as short pauses, those between the 25th and 75th percentiles as medium pauses, and those above the 75th percentile as long pauses. For improved visualization and normalization, the elapsed time t between action $a_{i}$ and $a_{i+1}$ was transformed using log(t), which better approximates a normal distribution. This thresholding approach aligns with Tenison and Arslan (2020), who also used the 25th and 75th percentiles to distinguish short, medium, and long pauses. However, their thresholds were fixed based on the overall pause-time distribution in their dataset and did not account for the frequency or timing characteristics of specific action pairs. In contrast, our proposed approach derives pause categories from the local time distribution of each action pair, incorporating both how often a pair occurs and the typical duration between the two actions. This yields pause classifications that are more sensitive to the structure of the underlying behavioral sequences.

**Figure 3:**
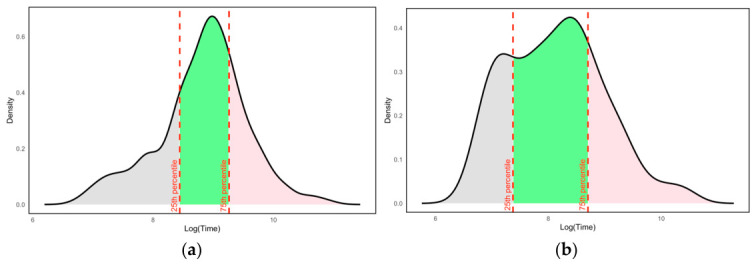
Illustration of “pause” action incorporated into the time-embedded n-gram model. The distribution of the logarithm of elapsed time between adjacent actions is divided using the 25th and 75th percentiles as thresholds. A *short pause* is defined as an interval below the 25th percentile (grey area), a *medium pause* as an interval between the 25th and 75th percentiles (green area), and a *long pause* as an interval above the 75th percentile (pink area). (**a**) An illustration of the log-time distribution for the action pair “Select I45 → Select I49” (399 occurrences); (**b**) An illustration of the distribution for “Select I53 → Select I54” (274 occurrences).

Figure 3 illustrates two examples of the distributions of elapsed time between action pairs. Based on the percentile thresholds, we assigned pause categories on elapsed time duration between each action pair, that is, the grey color area for short pauses (shorter than the 25th percentile), green for medium (between the 25th and 75th percentiles), and pink for long (longer than the 75th percentile). These time-embedded bigrams were then concatenated to form new action sequences for each participant.

Let A as an ordered action sequence consisting of n actions a, with elapsed time between actions as $t_{n-1}$, $A^{\prime }$ is the time-embedded sequence of A with a categorized time pause $p_{k}$ into three categories, short ($p_{k=s}$), medium ($p_{k=m}$), and long ($p_{k=l}$), pauses by the elapsed time distributions based on the specific pair of actions ($a_{n-1},\;a_{n})$.

(1) $$A=\{a_{1}\;t_{1}\to \;a_{2}\;t_{2}\to \;{\ldots a}_{i}\;t_{i}\to \;a_{i+1}\ldots \ldots a_{n-1}{\;t_{n-1}\to \;a}_{n})$$

(2) $$A^{\prime }=\left\{a_{1},p_{k},a_{2},p_{k},{\ldots a}_{i},p_{k},\ldots a_{n-1},p_{k},a_{n}\right\}$$

A concrete example can be illustrated with the sequence A = {Start (15 s), Select I44 (40 s), Select I58 (2 s), End}. From this sequence, we first extract the action pairs (bigrams): {Start, Select I44}, {Select I44, Select I58}, and {Select I58, End}. We then examine the frequency of each bigram across the dataset; because all three occur at least five times, none are removed. Next, we calculate the distribution of elapsed times for each action pair. For instance, the 15 s interval between *Start* and *Select I44* falls below the 25th percentile of that pair’s time-duration distribution, so it is categorized as a short pause, yielding the trigram {Start, Short Pause, Select I44}. The same procedure is applied to the remaining bigrams. Consequently, sequence A is transformed into $A^{\prime }\;=\;\{Start,\;Short\;Pause,\;Select\;I44,\;Long\;Pause,\;Select\;I58,\;Medium\;Pause,\;End\}$, with each pause category reflecting the local time distribution of its corresponding action pair.

To further enhance feature representation, we applied the TF-ISF weighting scheme (Q. He & von Davier, 2016) to the time-embedded sequences. Similar to the traditional n-grams, we treated “pause” action as an embedded action analogous to terms in natural language processing. A term frequency of a mini-sequence (with elapsed pause action) ${tf}_{i,j}\;$(gram i in sequence j) and its inverse sequence frequency ${ISF}_{i}=log(\frac{N}{sf_{i}})\geq 0$, where N indicates the total number of sequences in the collection, namely, the total number of test takers, 596, in this study, and ${sf}_{i}$ is the number of sequences where the mini-sequence i appears, are combined into a single weight as follows:

(3) $$weight\left(i,j\right)=\{\begin{array}{ll} \left[1+\log\left({tf}_{i,j}\right)\right]\log\left(\frac{N}{{sf}_{i}}\right) & if\;{tf}_{i,j}\geq 1\\ 0 & if\;{tf}_{i,j}=0 \end{array}$$

The first clause applies to grams occurring in the same sequence, whereas for grams that do not appear $\left({tf}_{i,j}=0\right)$, we use weight (i,j)=0. This weighting scheme highlights distinctive features by amplifying actions that are less frequent across sequences, thereby improving the discriminative power of the time-embedded representations.

In this study, we incorporated both n-grams (without elapsed time) and time-embedded n-grams as low-level sequential features to illustrate the contribution of time-embedded representations in the prediction model. The standard n-grams captured only adjacent action pairs, yielding 531 bigrams. In contrast, the time-embedded n-grams were constructed as trigrams that included at least one pause action, resulting in 1399 trigrams in total.

### 3.4. Aggregate-Level Process Indicators

In addition to the time-embedded n-grams extracted from the action sequences, we incorporated nine process indicators that were designed to aggregate low-level sequential features into more interpretable metrics as suggested by Goldhammer et al. (2021). These indicators include three time-related measures (i.e., total response time, time to the first action, and time to the final action before task completion), five count-based measures (i.e., total number of actions, number of final-response actions, number of help function uses, number of deselections, and number of off-map clicks), and one computational redundancy indicator. The redundancy ratio was defined as the number of final-response actions divided by the total number of actions to capture the extent to which respondents engaged in unnecessary steps. Values closer to 1 indicate that respondents used fewer redundant actions, and that most actions directly contributed to the final response. In contrast, values approaching 0 suggest longer, less efficient action sequences that did not directly support reaching the solution. Table 2 summarizes the nine aggregate-level process indicators and their corresponding interpretations.

### 3.5. Feature Selection and Analytic Strategy

Feature selection was conducted to differentiate adults with high versus low numeracy levels based on their interactions with the items. The process involved two steps. First, we applied chi-square feature selection to identify the time-embedded n-grams that most effectively distinguished between the binary groups (correct vs. incorrect, low numeracy vs. high numeracy). We then used ensemble-based feature selection to automatically identify the most robust predictors across both the aggregate-level indicators and the low-level sequential features derived from the time-embedded n-gram representations.

#### 3.5.1. Chi-Square Feature Selection

Following He and von Davier (2016), we applied chi-square feature selection, a method widely used in textual analysis for identifying highly discriminative features and comparing distributions across corpora (Manning & Schütze, 1999; see Forman, 2003, for additional models). Given the structural parallels between textual and process data, this approach is well suited for detecting actions or action vectors that effectively distinguish the two groups. For each action or action vector, we constructed a 2 × 2 contingency table crossing its presence or absence with group membership (i.e., high vs. low numeracy), as shown in Table 3. Let $n_{i}\;$and $m_{i}\;$denote the weighted frequencies of an action in groups $C_{1}$ and $C_{2}$, respectively, and let len(C) represent the total weighted action count within each group. The chi-square statistic tests whether action occurrence is independent of group membership; under the null hypothesis, the two groups should exhibit proportional action distributions. For a 2-by-2 contingency table, the chi-square value is computed as:

(4) $$\chi^{2}=\frac{M{\left(O_{11}O_{22}-O_{12}O_{21}\right)}^{2}}{\left(O_{11}{+O}_{12})(O_{11}{+O}_{21})(O_{12}{+O}_{22})(O_{21}{+O}_{22}\right)}$$

where M is the total number of actions in the collection, and $O_{ij}$ represents the weighted cell counts in the contingency table (Agresti, 1990; Bishop et al., 1975), namely $O_{11}$ and $O_{12}$ represent occurrences in the two groups and $O_{21}$ and $O_{22}$ represent non-occurrences. Actions with larger chi-square values are more informative for classification (Manning & Schütze, 1999). We therefore ranked all actions by their chi-square scores and treated the highest-scoring actions as robust discriminators. Using a 95% confidence threshold (χ^2^ > 3.84), we identified actions that occurred significantly more often in one group. When the ratio $n_{i}/m_{i}$ exceeded $len(C_{1})/len(C_{2})$, the action was considered more typical of group $C_{1}$ (a “positive indicator”); otherwise, it was more characteristic of group $C_{2}\;$(a “negative indicator”) following Oakes et al. (2001).

#### 3.5.2. Ensemble Machine Learning Feature Selection

The RF algorithm (Breiman, 2001), an extension of the Classification and Regression Tree (CART; Breiman et al., 1984), is an ensemble method that aggregates predictions from multiple decision trees to reduce variance and improve accuracy through randomized tree construction. Each tree is built on a bootstrap sample and, at every split, considers only a randomly selected subset of predictors to enhance model robustness (Dietterich, 2000; Han et al., 2019; Q. He et al., 2023b). Because RF relies on many decision trees, understanding the underlying tree-growing mechanism is essential. A decision tree recursively partitions the predictor space using greedy binary splits that minimize impurity (classification) or mean squared error (regression), producing increasingly homogeneous nodes as the tree deepens (Han et al., 2019). A key feature of RF is that, instead of searching all predictors for the optimal split, each node evaluates only a random subset of variables. This design enables RF to handle high-dimensional predictor spaces efficiently, mitigates data sparsity, and improves numerical stability.

In this study, we used binary splits (two child nodes per parent) and allowed trees to grow without explicit constraints on depth or minimum node size, using the default settings of the randomForest package (Liaw & Wiener, 2002) in R version 4.5.1. Our primary goal was to identify the optimal combination of *mtry* and *ntree* that maximized prediction accuracy. We randomly assigned 80% of the data to the training set and 20% to the testing set and implemented 10-fold cross-validation to ensure stable performance estimates. *mtry* values were selected based on the default value of $\sqrt{p}$ for the RF classifier, where p is the number of predictors. There are 530 and 1408 predictors from bigram and trigram features, respectively. Therefore, *mtry* values of 10, 20, and 30 were used to evaluate a reasonable range of smaller to moderate predictor sets at each split, while having a consistent tuning grid across bigrams and trigrams. We also tuned a wide range of *ntree* values from 100 to 500 to examine if model performance stabilized as the ensemble size increased. This procedure allowed us to assess variability due to random train-test splits and to compute the average accuracy and standard deviation across trials, providing a clearer and more reliable evaluation of RF performance across multiple random replications.

XGBoost (T. Chen & Guestrin, 2016) is an efficient and highly accurate implementation of gradient boosting machines, offering optimized computation and strong predictive performance (Acisli-Celik & Yesilkanat, 2023; H. Liu et al., 2022). The algorithm constructs an ensemble of weak learners, here, individual classification trees, and iteratively combines them to form a strong predictive model (H. Liu et al., 2022; Ulitzsch et al., 2023). Its objective function integrates a loss term and a regularization term, with the loss approximated through a second-order Taylor expansion. Regularization plays a central role in controlling model complexity and reducing overfitting, while each boosting iteration updates the model by minimizing the objective function and refining residual errors.

XGBoost includes several hyperparameters designed to prevent overfitting and manage missing values. The parameter *max_depth* limits the depth of each tree, balancing the ability to capture complex patterns with the risk of fitting noise. The parameter *min_child_weight* specifies the minimum sum of instance weights required for a split, preventing trees from growing too deep based on minor fluctuations in the data. Because boosting repeatedly transforms leaves into internal nodes until reaching the maximum depth (Ulitzsch et al., 2023), identifying an appropriate combination of these hyperparameters is essential for stable and generalizable model performance.

All analyses were conducted in R version 4.5.1 using the *xgboost* package (T. Chen et al., 2026), with hyperparameter tuning performed through *caret* (Kuhn, 2021). An 80/20 train–test split was applied, and a five-fold cross-validation procedure ensured reliable performance estimates. Hyperparameters were tuned using grid search with a fixed learning rate (eta = 0.01) and a fixed number of boosting rounds (nrounds = 100), to allow gradual learning and to keep computational cost manageable. Tree depth was tuned using *max_depth* values of 6, 8, and 10 to compare moderate to more complex tree structures. The *gamma* parameter was tuned using 0, 1, and 5 to control the minimum loss reduction required to make a split, with higher values meaning more conservativeness. The *colsample_bytree* and *subsample* parameters were tuned using values 0.5, 0.7, and 1, and 0.6, 0.8, and 1, respectively, to evaluate whether features and row subsampling improved generalization and reduced overfitting. Finally, *min_child_weight* values of 1, 3, and 5 were tuned to control the minimum amount of information required in child nodes.

In this study, we defined the prediction task as a four-category classification based on performance and numeracy level, as shown in Table 1: incorrect–low numeracy (IL), incorrect–high numeracy (IH), correct–low numeracy (CL), and correct–high numeracy (CH), with 196, 65, 100, and 235 participants, respectively. This framework allows us to examine behavioral and strategy differences across numeracy levels in both successful and unsuccessful problem-solving groups. Model performance was evaluated using four standard metrics, accuracy, precision, recall, and F1 score, to assess how effectively the selected features predicted respondents’ categories. We also analyzed variable importance from the ensemble model to identify the predictors that contributed most strongly to classification performance.

## 4. Results

### 4.1. Behavioral Patterns Between Correct and Incorrect Groups (RQ1)

To address RQ1, we first compared correct (N=335) and incorrect (N=261) groups to identify key features that may help explain their success or failure. Table 4 summarizes the aggregated process indicators for the correct and incorrect performers, further broken down by numeracy level. Focusing on the “correct” and “incorrect” columns, the time-related indicators show that the correct group spent an average of 107 s on the Map task, 11 s faster than the incorrect group. They also demonstrated quicker first-interaction times, with nearly a 10 s advantage. The time since the last interaction to the task completion did not differ significantly between groups.

For the count-related indicators, the correct group performed slightly fewer total actions than the incorrect group, but they executed more final-response actions. This pattern produced a higher redundancy ratio in the correct group, indicating that a greater proportion of their actions contributed directly to the final response and that they engaged in fewer redundant actions overall. No significant differences were observed in the use of the help function between successful and unsuccessful performers. Additionally, the correct group made fewer deselections and fewer off-map clicks, suggesting more focused and efficient interaction behavior.

We also compared the final-response actions between the successful and unsuccessful groups. Table 5 presents the most frequent solution patterns for the correct and incorrect performers. The key actions—I45, I49, I53, I54, and I58—formed the core sequence for correct solutions, with I44 appearing as an optional intermediate step. Notably, the correct group demonstrated a highly consistent problem-solving strategy: approximately 78% of respondents followed the same action order, with only minor variations involving I44. Average numeracy scores did not differ significantly across these correct-solution patterns, which aligns with our expectations, that is, once respondents identified the correct route, the specific order of correct steps did not depend on numeracy level.

In contrast, the incorrect group exhibited far more diverse incorrect strategies. Among the top incorrect patterns, many respondents ended with only a single remaining action, indicating uncertainty about how to approach the task. Nearly 30% chose an incorrect “left-turn” path, suggesting surface-level reasoning rather than a spatial strategy. Their most common incorrect sequences involved I45, I49, I52, I51, and I55. This frequent “left turn” misinterpretation likely reflects difficulty shifting between egocentric and allocentric spatial representations (Ekstrom & Hill, 2023; Moffat, 2009). To solve the task correctly, respondents needed to adopt an egocentric frame by imagining themselves moving along the route, while also coordinating that perspective with the fixed map layout as an allocentric representation. Many incorrect respondents appeared to rely instead on a surface-level interpretation of “left” as the literal left side of the screen, rather than the traveler’s left relative to their orientation. This suggests challenges with mental rotation and spatial transformation at the critical I49 → I53 turning point, where respondents must imagine themselves facing east and recognize that the correct left turn is visually represented on the right side of the map. These errors likely also increase working memory demands, as respondents must coordinate the route instructions, current orientation, and map layout while determining the next action.

Importantly, numeracy scores differed significantly depending on which incorrect strategy respondents used. For example, respondents whose final response consisted of only I58, the endpoint of the correct path, achieved an average numeracy score of 268.06, about sixty points higher than those whose sole final action was I45, the starting point of the correct path. Although both responses were incorrect, the ability to identify the correct endpoint reflects stronger underlying numeracy skills.

We further employed the chi-square feature selection method to identify the most informative sequential patterns distinguishing the correct and incorrect groups. This analysis incorporated both traditional n-grams (without elapsed time), specifically bigrams that capture adjacent action pairs, and time-embedded n-grams, specifically most of the trigrams that include at least one pause action to reflect whether respondents made short, medium, or long pauses between consecutive selections.

Table 6 summarizes all bigram and trigram actions associated with the two groups. The most robust features highlight that understanding how to “turn left” in a spatial dimension, rather than interpreting “left” literally on the screen, was the strongest discriminator between successful and unsuccessful solutions. The time-embedded *n*-gram results further illuminate this pattern: at the key turning point from I49 → I53, respondents in the correct group typically took a short or medium pause, suggesting confident spatial reasoning. In contrast, incorrect respondents showed longer pauses around I49 → I52 and I52 → I51 turning points, indicating greater cognitive uncertainty during spatial transformation.

Another notable robust feature involved identifying the correct starting point. Several of the strongest bigram features in the incorrect group involved selecting an incorrect starting location (e.g., I9, I18, I26, I37), corresponding to the orange point in Figure 2. These respondents failed from the outset because they misinterpreted the map’s symbols or indicators. Although the instructions clearly direct respondents to begin at the blue circle, individuals with lower skills may struggle to identify this correctly. As a result, some began at the orange circle, generating an entirely incorrect sequence of actions.

### 4.2. Robust Features in Respondents with High and Low Numeracy Levels (RQ2)

In Table 4, the High Numeracy and Low Numeracy columns summarize respondents’ behavioral patterns across numeracy groups. On average, the low-numeracy group spent 6 s longer completing the task than the high-numeracy group. Interestingly, respondents with higher numeracy spent 1.2 s more after making their final action before moving to the next question, suggesting that they may engage in additional verification before finalizing their response. The high-numeracy group also performed fewer total actions and made fewer deselections, indicating more decisive and efficient interaction. Their number of off-map clicks was substantially lower as well. The redundancy ratio further shows that high-numeracy respondents contributed a greater proportion of actions directly to the final response, meaning they engaged in fewer redundant actions during the problem-solving process.

Table 7 presents the prediction results for RF and XGBoost using both n-grams (without elapsed time) and time-embedded n-grams. Across the ensemble models, the indicators demonstrated solid predictive performance for numeracy. Both RF and XGBoost achieved accuracies above 70%, with RF performing slightly better overall. RF with time-embedded n-grams yielded marginally higher accuracy and precision than with bigrams alone, indicating that elapsed time contributes modestly to predictive power.

Both RF and XGBoost selected similar sets of robust features, particularly when time-embedded n-grams were combined with the nine process indicators. Among the top 10 features, the two models shared 8 out of 10, with only minor differences in ranking, which shows the stability of feature importance. Table 8 presents the top 10 variables contributing the most information to the prediction model. Notably, the time-related variables emerged as the most informative overall. Among the time-embedded n-grams selected by RF, the sequence I49 → medium pause → I53 stands out. This pattern likely reflects respondents’ effort to mentally evaluate the “left-turn” concept, demonstrating the use of spatial cognition during problem solving. Time to the last action captures the pause duration after respondents completed the route but before moving to the next item. This pause was significantly longer in the incorrect group (regardless of numeracy level) and in the high-numeracy group (regardless of correctness). Interestingly, the IH group had the longest pause out of all groups. A possible interpretation is that high-numeracy respondents might tend to double-check critical turning decisions, such as left versus right, and when they ultimately choose incorrectly, this may lead to greater reluctance before exiting the item. The IH group also exhibited the longest time to their first interaction and the longest total time on the Map task. This pattern suggests high engagement and deeper cognitive effort, but also the possibility of overthinking the instructions, which might have contributed to their incorrect responses. It is also noted that the CH group showed the highest redundancy ratio, indicating that they used the most precise and efficient sequence of actions to reach the correct solution, with minimal unnecessary steps.

## 5. Discussion

Spatial cognition encompasses a wide range of mental processes through which individuals acquire, represent, organize, and interpret spatial information. It enables people to navigate environments, manipulate objects, and communicate spatial relationships (Spence & Feng, 2010). Because of its multifaceted nature, spatial cognition spans several interrelated dimensions rather than a single definable construct. In this study, we examined one spatial item from PIAAC and leveraged process data to understand how adults approach a map-based spatial navigation task. By comparing successful and unsuccessful performers and high- and low-numeracy groups, we identified key behavioral and cognitive patterns that differentiated performance.

Success on this item required respondents to interpret the instruction “turn left” not as a literal left on the screen but as a spatially embedded left from the traveler’s perspective. This required respondents to shift between egocentric and allocentric spatial representations, moving from a self-centered frame of reference to the fixed environmental layout of the map (Ekstrom & Hill, 2023; Moffat, 2009). Such perspective-taking depends on mental rotation and spatial transformation, as respondents must mentally simulate their orientation while navigating the route (Hegarty, 2018; Uttal et al., 2013).

The frequent incorrect selection of the I49 → I52 → I51 → I55 pathway suggests that many respondents relied on a surface-level interpretation of direction rather than this embodied spatial reasoning process. Longer pauses at these turning points further indicate increased cognitive effort and likely reflect working memory demands, as respondents must temporarily maintain route instructions, current orientation, and destination goals while evaluating the next step (Burgess, 2008; Ekstrom & Hill, 2023). These findings support embodied cognition theory by suggesting that successful numeracy performance in digital spatial tasks depends not only on mathematical reasoning, but also on the ability to coordinate perception, movement, and spatial representation in real time (Barsalou, 1999; Z. Hawes & Ansari, 2020; Mix & Cheng, 2012; Wilson, 2002).

The process indicators and sequential features further revealed differences in how numeracy levels shape problem-solving behavior. Consistent with prior research on cognitive efficiency (Q. He et al., 2021), the high-numeracy group demonstrated more streamlined interaction patterns: shorter task durations, fewer deselections, fewer off-map clicks, and a higher redundancy ratio, indicating that a larger share of their actions directly contributed to the final solution. These behaviors suggest more deliberate and goal-directed strategies, with less exploratory or corrective behavior during problem solving.

Beyond the specific navigation patterns observed in the task, the machine-learning results further highlight the central role of temporal and sequential dynamics in differentiating performance. Across RF and XGBoost, the most informative predictors consistently included total response time, time to first interaction, and time to last action. The addition of time-embedded n-grams provided modest but meaningful improvements, suggesting that pauses and temporal spacing may capture meaningful aspects of respondents’ cognitive processes during spatial navigation, particularly during key directional decision points (e.g., the transition from I49 to I53, where respondents must mentally evaluate the left-turn instruction from an embodied spatial perspective). Together, these findings underscore the potential value of process data for understanding how adults engage with spatially demanding digital numeracy tasks.

At the same time, caution is warranted when interpreting pauses as indicators of cognitive load, hesitation, or other internal cognitive states. As Tenison and Arslan (2020) noted, “different points throughout an educational activity are indicative of different cognitive activities,” which underscores the ambiguity inherent in pause-based inference. Although prior work has shown that pauses between actions can provide informative signals about student proficiency (e.g., Dang et al., 2017; Pelánek, 2014), their interpretation is not uniform. Research that uses pauses to identify wheel-spinning, gaming the system, or productive persistence typically relies on expert qualitative coding and detailed knowledge of the tutor environment to determine when a pause is likely to reflect these behaviors (Aleven et al., 2004; Paquette et al., 2014). Such approaches are informative but depend heavily on human judgment and are generally applied in systems with a constrained action space.

Another example of this ambiguity arises in the pause between the task starting and the first action. A short pause may indicate that a student skipped reading the instructions, made a rapid guess, or simply read very quickly (Wise, 2017). Relying solely on pause duration in this context does not provide a complete picture. Thus, while pauses may reflect meaningful aspects of problem-solving behavior, these interpretations require interpretation within the broader task context rather than as isolated indicators of cognitive processing.

### 5.1. Pedagogical Implications for Adults’ Numeracy Instruction

A broader consideration in interpreting these results is the strong interdependence between literacy and numeracy skills. PIAAC data show a robust correlation between the two domains (r ≈ 0.883 across countries; OECD, 2016). As the PIAAC framework notes, numeracy tasks often require reading comprehension, literacy strategies, and prior literacy experiences (OECD, 2012). This interdependence is particularly relevant for the current findings, as successful performance on the Map item required respondents to correctly interpret the instruction “turn left” from a spatially embedded perspective rather than a screen-based orientation. Misinterpretations at this stage may contribute to longer response times, delayed initial interactions, or hesitation before finalizing a response, which were patterns observed among lower-performing or incorrect groups. In digital environments, where numeracy tasks are embedded in textual instructions and layered visual representations, adults with limited literacy may struggle to decode task requirements (e.g., misunderstanding the starting point on the map) regardless of mathematical reasoning. Thus, disentangling the role of literacy in digital numeracy performance is essential for designing equitable assessments and interventions. This distinction becomes especially important in adult education settings, where learners must interpret task instructions while simultaneously engaging with mathematical and spatial demands.

In the U.S., adults with low numeracy performance are often enrolled in adult foundational education programs to build literacy, numeracy, social studies, science, and writing skills to complete a high school equivalency degree (Lesgold & Welch-Ross, 2012). Although numeracy is a key competency, many of these programs seem to emphasize the skills to pass the GED assessment more than the purpose of mathematical concepts in daily life (Curry, 2019). Curry (2019) provides several real-life examples of how adult education instructors can use real-world math examples (e.g., nutritional labels) and scaffold instruction to adjust the difficulty level for learners at varying proficiency levels. In addition, assessments and real-life demands are increasingly digital, and it is important to address digital numeracy skills in adult foundational education (Alamprese & Hoogland, 2025). These instructional challenges are particularly relevant to tasks like the PIAAC Map item, where success depends not only on mathematical reasoning but also on interpreting spatial instructions and navigating digital environments.

More broadly, these findings point to the importance of supporting spatial reasoning as a core component of numeracy instruction. A meta-analysis examining spatial skills across the lifespan reported that these skills are malleable and that training programs lead to improvements in spatial abilities (Uttal et al., 2013). Specific to adults with low numeracy, adult foundational education programs can integrate different approaches to increase spatial cognition and use embodied approaches. For example, Tighe et al. (2021) suggest using manipulatives (e.g., blocks) to ground abstract mental rotation and concepts. Programs can also embed math concepts in everyday spatial activities (reading a map, cooking and measuring to show fractions and amounts with kitchen utensils; Tighe et al., 2021; X. Wang et al., 2017).

Process-based indicators may also help educators better understand how adults engage with digital numeracy tasks. Such dynamic behavioral indicators offer meaningful opportunities to track test-takers’ strategies and engagement patterns, thereby supporting evidence-based instructional decisions. Integrating these process-based insights into pedagogical practice can strengthen instructional guidance and promote more targeted learning support.

### 5.2. Limitations

First, we analyzed only one spatial numeracy item, as PIAAC includes only a single map-based task involving spatial navigation. Although the sample is nationally representative, findings from a single item should be interpreted cautiously and cannot be assumed to represent the full construct of numeracy or spatial cognition. The Map task primarily reflects the dimension and shape domain within the PIAAC numeracy framework, which includes spatial orientation, location, measurement, and interpretation of two- and three-dimensional representations (OECD, 2012, 2016). Successful performance also depended on respondents’ ability to shift between egocentric and allocentric spatial representations when interpreting directional instructions such as “turn left” (Moffat, 2009; Ekstrom & Hill, 2023). As a result, our findings are most directly generalizable to spatial reasoning demands embedded within numeracy tasks, rather than to numeracy more broadly.

At the same time, some observed behaviors may be more specific to the unique design of this item. In particular, the left-turn misinterpretation at the I49 → I53 turning point likely reflects a task-specific challenge tied to the map layout and directional perspective required by this assessment. In contrast, other process indicators such as pause duration, response timing, hesitation before finalizing a response, and redundancy in action sequences may reflect broader problem-solving behaviors that are transferable across other digital numeracy and problem-solving assessments (Goldhammer et al., 2014, 2017; Q. He et al., 2021).

The characteristics of the Map item itself also shape how broadly these findings can be interpreted. The Map task is considered a Level 3 difficulty item, with a discrimination parameter of 1.067 and a difficulty parameter of 0.653 (OECD, 2019, p. 627). As a mid-level difficulty task, it allowed us to include a broad distribution of both high- and low-numeracy adults. Although 16 items (29% of total numeracy items) in PIAAC Cycle 1 represent the dimension and shape content area, the Map item is unique because it is the only one that combines spatial navigation with digital process data collection. Future assessment design would benefit from including multiple items with similar spatial navigation demands rather than relying on a single task, allowing researchers to determine whether these behavioral patterns remain stable across comparable contexts and levels of difficulty. Including additional map-based or adaptive problem-solving items, such as the PIAAC Cycle 2 “Dinner Preparation” task (Figure 1), would strengthen validation and provide a more comprehensive picture of adults’ spatial cognition across assessment contexts. Multimodal process data, such as eye-tracking, could further support this work by providing richer evidence of how adults allocate attention and coordinate spatial reasoning during task completion (Kiefer et al., 2017).

Second, PIAAC also offers a rich background questionnaire, which could be used to link behavioral patterns to demographic variables, ICT skills, and educational experiences. These analyses may offer a valuable avenue for future work to develop adult instruction that is customized to the needs of specific groups. Beyond the studies in PIAAC Cycle 1, future work could make demographic and skill-based comparisons between the two cycles.

Existing findings from PIAAC Cycles 1 and 2 already demonstrate substantial demographic differences in numeracy performance, including race/ethnicity, such that adults self-identifying as White have higher average performance than those identifying as Black, Hispanic, or Other, with these groups disproportionally over-represented in the lowest proficiency levels (Level 1 and below). There are also differences by educational attainment, such that adults with a high school degree or higher have significantly higher average performance than adults with less than a high school education; in particular, those with less than a high school education represent 58–65% of at or below Level 1. Native-born adults have an average higher performance than non-native adults. Males also tend to have better average numeracy performance than females. Adults self-reporting excellent or very good overall health exhibit higher average numeracy performance than those reporting fair or poor health. Finally, employed adults have a higher average numeracy performance than those who are unemployed or out of the work force. Interestingly, there are limited differences in average numeracy performance by 10-year age bands (National Center for Education Statistics, 2024). Thus, future cross-cycle comparisons may help better understand how numeracy performance is tied to demographic considerations.

Third, the pause action could be expanded beyond the three categories (short, medium, long) used in this study. Researchers may define and justify finer-grained thresholds—for example, dividing time intervals into ten bins instead of three—to capture more detailed patterns of time use. However, such granularity introduces a trade-off: smaller time bins may occur too infrequently to be reliably incorporated into bigrams. In addition, trigrams that include two actions with a pause in between are likely to yield more meaningful behavioral information than sequences containing two pause categories surrounding a single action. Future research could explore these design choices more systematically and evaluate their impact on predictive performance.

Finally, because the overall sample size in this study was relatively small and the degree of imbalance across outcome categories was not substantial, no over- or under-sampling techniques were applied. However, when prediction tasks involve more pronounced imbalance, the use of appropriate oversampling or under-sampling methods is recommended to justify the sample size in each category, as imbalance can meaningfully influence predictive performance.

## 6. Conclusions

This study highlights the importance of using process data to gain deeper insight into spatial cognition processes and problem-solving behaviors. The time-embedded n-grams approach provides a simple yet powerful way to integrate multidimensional sequence data with temporal information, offering a more nuanced understanding of how individuals engage with complex, digitally mediated tasks. These insights are especially relevant for adult learners, where performance may reflect not only domain knowledge but also the ability to interpret instructions and navigate task environments.

From a methodological perspective, these findings also underscore the value of approaches that capture temporal dynamics in task engagement. We recommend applying both n-grams and time-embedded n-grams methods in future analyses to assess whether elapsed time contributes meaningfully to predictive performance. When prediction results differ substantially between the two approaches, it may signal that elapsed time is a critical cognitive factor that should not be overlooked, particularly in designing assessments and supports for adult learners.

## Figures and Tables

**Table 1:** Statistical description of the U.S. sample (*N* = 596).

	*N*	Gender (%)		Race (%)				Age (Years)		ICT at Home		ICT at Work	
		Female	Male	White	Black	Hispanic	Others	*M*	*SD*	*M*	*SD*	*M*	*SD*
Low-Numeracy	296	62.50	37.50	62.93	16.33	10.88	9.86	37.58	14.24	2.04	0.97	1.98	1.11
Correct	100	63.00	37.00	66.67	10.10	13.13	10.10	35.32	13.40	1.92	0.91	1.99	1.21
Incorrect	196	62.24	37.76	61.03	19.49	9.74	9.74	38.73	14.56	2.10	1.00	1.98	1.06
High-Numeracy	300	47.33	53.67	81.33	4.33	5.33	9.00	40.43	13.55	2.47	0.78	2.34	1.05
Correct	235	46.38	53.62	82.55	4.68	3.83	8.94	39.67	13.18	2.45	0.75	2.36	1.07
Incorrect	65	50.77	49.23	76.92	3.08	10.77	9.23	43.20	14.58	2.52	0.86	2.24	0.97
Correct	335	51.34	48.66	77.84	6.29	6.59	9.28	38.37	13.37	2.30	0.84	2.28	1.11
Incorrect	261	59.39	40.61	65.00	15.38	10.00	9.62	39.84	14.66	2.21	0.98	2.07	1.03
Total	596	54.87	45.13	72.22	10.27	8.08	9.43	39.02	13.96	2.26	0.90	2.20	1.09

Note. Race, ICT at Home and ICT at Work have omitted 2, 46, and 214 missing values, respectively. *M =* Mean, *SD =* standard deviation.

**Table 2:** Aggregate-level process indicators and interpretations.

Process Indicator	Category	Meaning
Total response time	Time	The total amount of time a respondent spent on the item from entry to final submission.
Time to the first action	Time	The elapsed time between item entry and the respondent’s first recorded action.
Time to the last action before task completion	Time	The elapsed time between the respondent’s last recorded action and item exit.
Total number of actions	Count	The total number of recorded interactions performed on the item.
Number of final-response actions	Count	The number of actions recorded in the final response.
Number of help function uses	Count	The total count of times the respondent clicked on the help function button.
Number of deselections	Count	The total count of times that a previously selected route was deselected.
Number of off-map clicks	Count	The number of clicks recorded outside the designated map area.
Redundancy ratio	Computation	The ratio of final-response actions to total actions, indicating the proportion of actions directly contributing to the final response.

Note. The total number of actions and the number of final-response actions represent two distinct concepts. The number of final-response actions is always less than or equal to the total number of actions.

**Table 3:** 2-by-2 contingency table for action i in chi-square score computation.

	$C_{1}$	$C_{2}$
action i	$n_{i}$	$m_{i}$
¬action i	${len\;\left(C_{1}\right)-n}_{i}$	${len\;\left(C_{2}\right)-m}_{i}$

Note. $C_{1}$ and $C_{2}$ represent the two study groups (i.e., high and low-numeracy groups). $n_{i}$ and $m_{i}$ indicate the weighted frequency of the action i occurs in $C_{1}$ and $C_{2}$ respectively. len(C) indicates the sum of the weighted action occurrences in each group.

**Table 4:** Distribution of process indicators by performance groups.

	Correct		Incorrect		High Numeracy		Low Numeracy	
	Mean	SD	Mean	SD	Mean	SD	Mean	SD
Total response time	107.48	44.92	118.76	69.57	109.45	54.23	115.42	60.12
Time to the first action	54.55	31.58	63.83	53.90	58.63	46.14	58.60	39.69
Time to the last action before task completion	7.43	8.34	7.80	12.39	8.20	9.34	6.98	11.17
Total number of actions	7.44	3.61	7.79	6.06	7.17	3.71	8.03	5.74
Number of final-response actions	5.38	0.49	4.70	2.34	5.24	0.91	4.92	2.11
Number of help function uses	0.01	0.11	0.01	0.11	0.02	0.13	0.01	0.08
Number of deselections	0.70	1.42	1.16	2.40	0.69	1.52	1.12	2.25
Number of off-map clicks	0.60	1.26	0.71	1.23	0.51	0.97	0.79	1.47
Redundancy ratio	0.82	0.22	0.73	0.27	0.82	0.22	0.73	0.26

Note. The time-related indicators are in the unit of seconds.

**Table 5:** Top Strategy patterns of final-response actions by correct and incorrect groups.

	Final Response Actions	Number of Respondents	Average Numeracy Score
Correct	I45|I49|I53|I54|I58	174 (51.9%)	292.5
	I45|I44|I49|I53|I54|I58	35 (10.4%)	283.61
	I45|I49|I54|I53|I58	24 (7.2%)	294.72
	I44|I45|I49|I53|I54|I58	17 (5.1%)	314.25
	I45|I49|I44|I53|I54|I58	14 (4.2%)	287.4
	I45|I49|I54|I53|I44|I58	10 (3.0%)	313.1
	I45|I49|I54|I53|I58|I44	7 (2.1%)	311.72
Incorrect	I45|I49|I52|I51|I55	58 (22.2%)	264.66
	I58	12 (4.6%)	268.06
	I45|I44|I49|I52|I51|I55	11 (4.2%)	249.45
	I45|I49|I51|I52|I55	9 (3.4%)	241.96
	I55	8 (3.1%)	238.35
	I45|I49|I54|I58	6 (2.3%)	248.26
	I45	5 (1.9%)	209.67

Note. Percentages are calculated conditional on each respective group. Correct group N = 335, incorrect group N = 261.

**Table 6:** Top 10 robust sequential features from n-grams and time-embedded n-grams in the correct and incorrect groups derived from the chi-square feature selection method.

	Rank	N-Grams (Bigrams)	$\chi^{2}$	Time-Embedded N-Grams (Trigrams)	$\chi^{2}$
Correct	1	Select I54, Select I58	215.93	Select I54, Medium Pause, Select I58	155.59
	2	Select I53, Select I54	214.51	Select I53, Medium Pause, Select I54	146.56
	3	Select I49, Select I53	190.64	Select I49, Medium Pause, Select I53	132.60
	4	Select I58, End	161.52	Select I54, Short Pause, Select I58	126.12
	5	Select I54, Select I53	120.69	Select I53, Short Pause, Select I54	124.78
	6	Select I53, Select I58	97.47	Select I58, Short Pause, End	123.13
	7	Select I49, Select I54	67.50	Select I58, Medium Pause, End	107.33
	8	Select I58, Select I44	59.14	Select I58, Long Pause, End	95.88
	9	Select I44, Select I58	55.03	Select I49, Short Pause, Select I53	92.91
	10	Select I53, Select I44	51.49	Select I53, Long Pause, Select I54	82.14
Incorrect	1	Select I55, End	133.11	Select I55, Medium Pause, End	120.16
	2	Select I51, Select I55	111.80	Select I51, Medium Pause, Select I55	103.89
	3	Select I52, Select I51	92.05	Select I52, Medium pause, Select I51	90.49
	4	Select I49, Select I52	76.89	Select I55, Short Pause, End	78.67
	5	Select I18, Select I9	43.33	Select I55, Long Pause, End	76.49
	6	Select I26, Select I18	43.33	Select I49, Medium Pause, Select I52	72.63
	7	Select I37, Select I26	37.21	Select I52, Long Pause, Select I51	69.7
	8	Deselect I47, End	30.56	Select I51, Short Pause, Select I55	67.34
	9	Select I47, Select I37	30.56	Select I49, Long Pause, Select I52	57.75
	10	Select I51, Select I47	30.56	Select I51, Long Pause, Select I55	51.92

**Table 7:** Prediction performance of Random Forest and XGBoost models with n-gram and time-embedded n-gram features.

Metric	N-Grams (Bigrams)		Time-Embedded N-Grams (Trigrams)	
	RF	XGBoost	RF	XGBoost
Accuracy	0.715 (0.027)	0.705 (0.034)	0.718 (0.028)	0.708 (0.042)
Recall	0.632 (0.051)	0.653 (0.072)	0.612 (0.048)	0.683 (0.046)
Precision	0.756 (0.043)	0.729 (0.048)	0.774 (0.045)	0.720 (0.059)
F1	0.689 (0.033)	0.686 (0.043)	0.682 (0.034)	0.699 (0.037)

Note. The results are based on 10-fold cross-validation and report the mean and standard deviation of the evaluation metrics.

**Table 8:** Top 10 features extracted from random forest with n-grams.

Rank	Extracted Features	Importance	IL		IH		CL		CH	
			Mean	SD	Mean	SD	Mean	SD	Mean	SD
1	Time to the final action	7.77	7.40	12.78	9.02	11.13	6.15	7.02	7.97	8.79
2	Total response time	6.77	116.57	65.25	125.35	81.43	113.17	48.76	105.06	43.07
3	Select I58, end	6.33	0.02	0.07	0.04	0.09	0.08	0.04	0.10	0.04
4	Number of final-response actions	6.16	4.67	2.53	4.78	1.65	5.41	0.49	5.36	0.48
5	Time to the first action	6.00	60.74	43.35	73.13	77.19	54.40	31.05	54.62	31.87
6	Total number of actions	4.81	8.10	6.54	6.88	4.23	7.89	3.72	7.25	3.56
7	Select I53, Select I54	4.19	0.01	0.04	0.01	0.04	0.10	0.07	0.11	0.07
8	Redundancy ratio	4.18	0.71	0.28	0.79	0.24	0.78	0.22	0.83	0.21
9	Select I54, Select I58	3.64	0.01	0.04	0.02	0.05	0.11	0.07	0.11	0.07
10	Select I49, Select I53	3.16	0.02	0.05	0.02	0.07	0.10	0.09	0.11	0.09

Note. The units for all time-related variables are expressed in seconds, and the values for bigram features represent their TF–ISF weights.

## Data Availability

The data used in this study come from the restricted PIAAC Cycle 1 dataset, which is available through the OECD upon approved access. The public-use file containing response data for the Map item can be downloaded directly from the OECD website. Further details are provided at: https://www.oecd.org/en/data/datasets/piaac-1st-cycle-database.html (accessed on 15 May 2026).

## Note

1 The scores on the numeracy domain ranged from 0–500, with a breakdown of proficiency levels as follows: Below Level 1 (0–175; lowest); Level 1 (176–225); Level 2 (226–275); Level 3 (276–325); Level 4 (326–375); and Level 5 (376–500; highest). Given concerns about the small number in the extreme groups, we set the higher numeracy group as Level 3 and above, and the lower numeracy group as Level 2 and below.

## Abbreviations

The following abbreviations are used in this manuscript (by the order of first occurrence):

PIAAC	Programme for the International Assessment of Adult Competencies
PISA	Programme for International Student Assessment
APS	Adaptive problem-solving
RF	Random forest
XGBoost	Extreme Gradient Boosting
CART	Classification and Regression Tree
IH	Incorrect high numeracy group
IL	Incorrect low numeracy group
CH	Correct high numeracy group
CL	Correct low numeracy group
